# Day-to-day blood pressure variability and dementia

**DOI:** 10.18632/oncotarget.22993

**Published:** 2017-12-06

**Authors:** Tomoyuki Ohara, Emi Oishi, Toshiharu Ninomiya

**Affiliations:** Department of Neuropsychiatry, Graduate School of Medical Sciences, Kyushu University, Fukuoka, Japan; Department of Epidemiology and Public Health, Graduate School of Medical Sciences, Kyushu University, Fukuoka, Japan

**Keywords:** dementia, Alzheimer’s disease, blood pressure

Dementia has become a worldwide public health and social care priority, as the number of patients with dementia has been rising mainly due to aging of population [1]. Blood pressure is well known to mark fluctuations in the short-term (24 hours), mid-term (days/week), and long-term (years), and recent evidence suggests that blood pressure variability (BPV) contributes significantly to the development and progression of cardiovascular disease [2]. Recently, several observational studies have reported that higher visit-to-visit BPV, independent of the average blood pressure level, is associated with a higher risk of cognitive impairment and dementia [3-6]. However, most of these studies were based on visit-to-visit BPV assessed by office blood pressure measurement, and no studies have investigated the association between day-to-day BPV assessed by home blood pressure measurement and risk of developing dementia. In a recent study published in *Circulation*, we assessed the association of day-to-day BPV on a home blood pressure basis with the risk of dementia in a prospective study of an elderly Japanese population [7].

In this study, a total of 1,674 community-dwelling Japanese elderly without dementia, aged 60 years or older, were followed up for 5 years (2007 to 2012). All participants measured their blood pressure in the morning for a median of 28 days (range, 3–28 days) using a validated digital electronic blood pressure device. Day-to-day systolic (SBP) and diastolic blood pressure (DBP) variabilities were defined with the coefficients of variation (CoV), calculated as the standard deviation of the mean blood pressure divided by the mean blood pressure times 100, and were categorized into quartiles. Dementia diagnoses were adjudicated by expert stroke physicians and psychiatrists and were based on rigorous evaluation of clinical information, neuroimaging, and autopsy findings. During the 5-year follow-up period, 194 patients developed all-cause dementia, of whom 47 had vascular dementia (VaD) and 134 had Alzheimer’s disease (AD).

The age- and sex-adjusted incidences of all-cause dementia, VaD, and AD increased significantly with increasing CoV levels of home SBP (all *P* for trend <0.05). These associations remained significant even after adjustment for potential confounding factors, including mean home SBP values. Compared with subjects in the first quartile of CoV levels of home SBP, the risks of all-cause dementia, VaD, and AD increased significantly in those in the fourth quartile (HR=2.27, *P*<0.001 for all-cause dementia; HR=2.79, *P*=0.03 for VaD; HR=2.22, *P*<0.001 for AD). Similar associations were observed for CoV levels of home DBP. When dividing the subjects into 4 groups according to the status of hypertension based on home SBP values and the CoV values of home SBP, both higher day-to-day BPV and home SBP values were significantly associated with the risk of VaD, whereas the risk of AD increased significantly in subjects with higher BPV irrespective of the home SBP values. There was no interaction between home SBP levels and CoV levels of home SBP on the risk of each subtype of dementia.

Previous observational studies have demonstrated a significant association between cognitive decline and long-term BPV, mainly assessed by monthly or yearly blood pressure measurements in the office [3-5], and only the Three-City Study reported a positive association between long-term visit-to-visit systolic BPV measured in the office and risk of all-cause dementia and AD, but not VaD [6]. The present study is thus the first to demonstrate a significant association between increased day-to-day BPV on a home blood pressure basis and the development of all-cause dementia, VaD, and AD, regardless of average home blood pressure. These findings raise the possibility that BPV may play an important role in the development of dementia. Meanwhile, there might be an important difference in the blood pressure-related pathological processes between VaD and AD: both higher BPV and higher mean SBP values were associated with VaD, but only higher BPV was associated with AD. These findings raise the possibility that increased BPV is a causative factor for VaD through the alterations in brain structure and function, while it is a marker of neurodegeneration and autonomic dysfunction in AD. However, since there has been no clinical trial to assess the association between reduction in BPV values and risk of dementia, any clinical trials addressing the effect of BPV modification on the risk of dementia would likely help to elucidate the blood pressure-related pathological processes underlying the development of dementia subtypes.

BPV has been known to promote oxidative stress, endothelial injury, impairment of vascular smooth muscle function, and vascular stiffening [8]. Therefore, one possible mechanism is that high BPV may promote dysregulation of the cerebral vasculature and directly cause alterations in brain structure and function, which could then lead to the development of dementia [7]. Another possible mechanism is that BPV may be a marker of neurodegeneration, which has specific findings in AD [7]. However, at present, since the exact mechanisms responsible for these associations are unclear, further studies are required to clarify whether day-to-day BPV is an indicator of future dementia or an interventional target for the prevention of dementia.

The novel finding of this study is that increased day-to-day blood pressure variability, independent of average home blood pressure, is a significant risk factor for the development of dementia, suggesting that measurement of day-to-day BPV on a home blood pressure basis may be useful for assessing future risk of dementia. If this finding were to be confirmed in clinical trials or larger cohort studies, BPV modification could become an effective medical strategy to reduce the burden of dementia.
